# ScFv Anti-Heparan Sulfate Antibodies Unexpectedly Activate Endothelial and Cancer Cells through p38 MAPK: Implications for Antibody-Based Targeting of Heparan Sulfate Proteoglycans in Cancer

**DOI:** 10.1371/journal.pone.0049092

**Published:** 2012-11-09

**Authors:** Helena C. Christianson, Toin H. van Kuppevelt, Mattias Belting

**Affiliations:** 1 Department of Clinical Sciences, Section of Oncology, Lund University and Skåne University Hospital, Lund, Sweden; 2 Department of Biochemistry, Nijmegen Centre for Molecular Life Sciences, Nijmegen, The Netherlands; 3 Skåne University Hospital and Oncology Clinic, Lund, Sweden; Medical College of Wisconsin, United States of America

## Abstract

Tumor development requires angiogenesis and anti-angiogenic therapies have been introduced in the treatment of cancer. In this context, heparan sulfate proteoglycans (HSPGs) emerge as interesting targets, owing to their function as co-receptors of major, pro-angiogenic factors. Accordingly, previous studies have suggested anti-tumor effects of heparin, *i.e.* over-sulfated HS, and various heparin mimetics; however, a significant drawback is their unspecific mechanism of action and potentially serious side-effects related to their anticoagulant properties. Here, we have explored the use of human ScFv anti-HS antibodies (αHS) as a more rational approach to target HSPG function in endothelial cells (ECs). αHS were initially selected for their recognition of HS epitopes localized preferentially to the vasculature of patient glioblastoma tumors, *i.e.* highly angiogenic brain tumors. Unexpectedly, we found that these αHS exhibited potent pro-angiogenic effects in primary human ECs. αHS were shown to stimulate EC differentiation, which was associated with increased EC tube formation and proliferation. Moreover, αHS supported EC survival under hypoxia and starvation, *i.e.* conditions typical of the tumor microenvironment. Importantly, αHS-mediated proliferation was efficiently counter-acted by heparin and was absent in HSPG-deficient mutant cells, confirming HS-specific effects. On a mechanistic level, binding of αHS to HSPGs of ECs as well as glioblastoma cells was found to trigger p38 MAPK-dependent signaling resulting in increased proliferation. We conclude that several αHS that recognize HS epitopes abundant in the tumor vasculature may elicit a pro-angiogenic response, which has implications for the development of antibody-based targeting of HSPGs in cancer.

## Introduction

The progression from malignant transformation into manifest tumor development requires the recruitment of blood vessels, *i.e.* the angiogenic switch [1], [2]. Angiogenesis is a multi-step process dependent on endothelial (EC) proliferation, migration into the surrounding tissue and finally differentiation into a new vessel. Binding of several angiogenic factors, *e.g.* VEGF-A, FGF, and HB-EGF to heparan sulfate (HS) polysaccharide chains underlines the importance of HS proteoglycans (PGs) in EC biology [3]–[5]. HSPGs act as co-receptors that present growth factors to their high-affinity tyrosine kinase signaling receptors at the cell surface [6], [7]. Extensive postsynthetic modifications of the linear HS chains, composed of repetitive N-acetylglucosamine and glucuronic acid disaccharide units, include sulfation at various positions along the chain, which results in negatively charged domains that provide binding sites for various growth factors, proteases and cytokines involved in tumor development [3]–[7]. Accordingly, mouse embryos engineered to express a VEGF-A lacking the HS-binding sequence exhibited a decrease in capillary branch formation [8], and transgenic mouse models with either a homozygous deletion of the HS-binding motif in PDGF-BB or reduced HS sulfation, displayed defective pericyte recruitment and obscured microvascular anatomy [9], [10]. Altered expression of regulatory enzymes involved in shaping the fine structure of HS chains have been implicated in cancer, *e.g.* gene hypermethylation mediated gene silencing of 3-O sulfotransferase and HSulf-1 was found in several cancers [11], and HSulf-2 over expression correlated with increased angiogenesis and growth factor binding capacity in breast cancer [12]. Further, HS-degrading heparanase has been associated with tumor progression and poor patient outcome through remodeling of the tumor microenvironment [13]. HSPGs have thus been implicated in several aspects of tumor development and angiogenesis; however, the role of HSPG as a target of anti-angiogenesis treatment remains to be defined. In fact, several strategies have been explored in order to achieve this purpose, most importantly the HS mimic heparin and its derivatives [14]–[18]. Major drawbacks with these compounds are their relative unspecific modes of targeting, and significant risks of bleeding complications associated with their anticoagulant activities. Other possible HS-targeted treatments for cancer therapy involve proteins or peptides with positively charged amino acid residues that act as competitive inhibitors of growth factor binding to HS chains [19].

Antibody-based therapy is one of the fastest growing therapy areas in medical oncology and antibodies targeting VEGF, the epidermal growth factor receptor 2 (HER-2), EGF receptor or CD20 have been approved in the treatment of *e.g.* metastatic breast and colorectal cancer and aggressive B-cell lymphomas [20]. Smaller recombinant antibodies like single-chain variable fragment (ScFv) antibodies are interesting options of improved targeting due to their favorable pharmacokinetics [21]–[23]. Interestingly, van Kuppevelt and co-workers have previously described the development and characterization of several epitope specific, phage display derived, ScFv αHS to probe the structural diversity of HS chains in various tissues [24], [25]. In the present study, we sought to identify ScFv αHS that recognize HS epitopes expressed in the vasculature of patient glioblastoma tumors, and further investigated the use of these αHS as a putative strategy to inhibit various aspects of EC function.

## Results

### Identification of αHS Clones that Recognize HS Epitopes of the Tumor Vasculature

**Figure 1:**
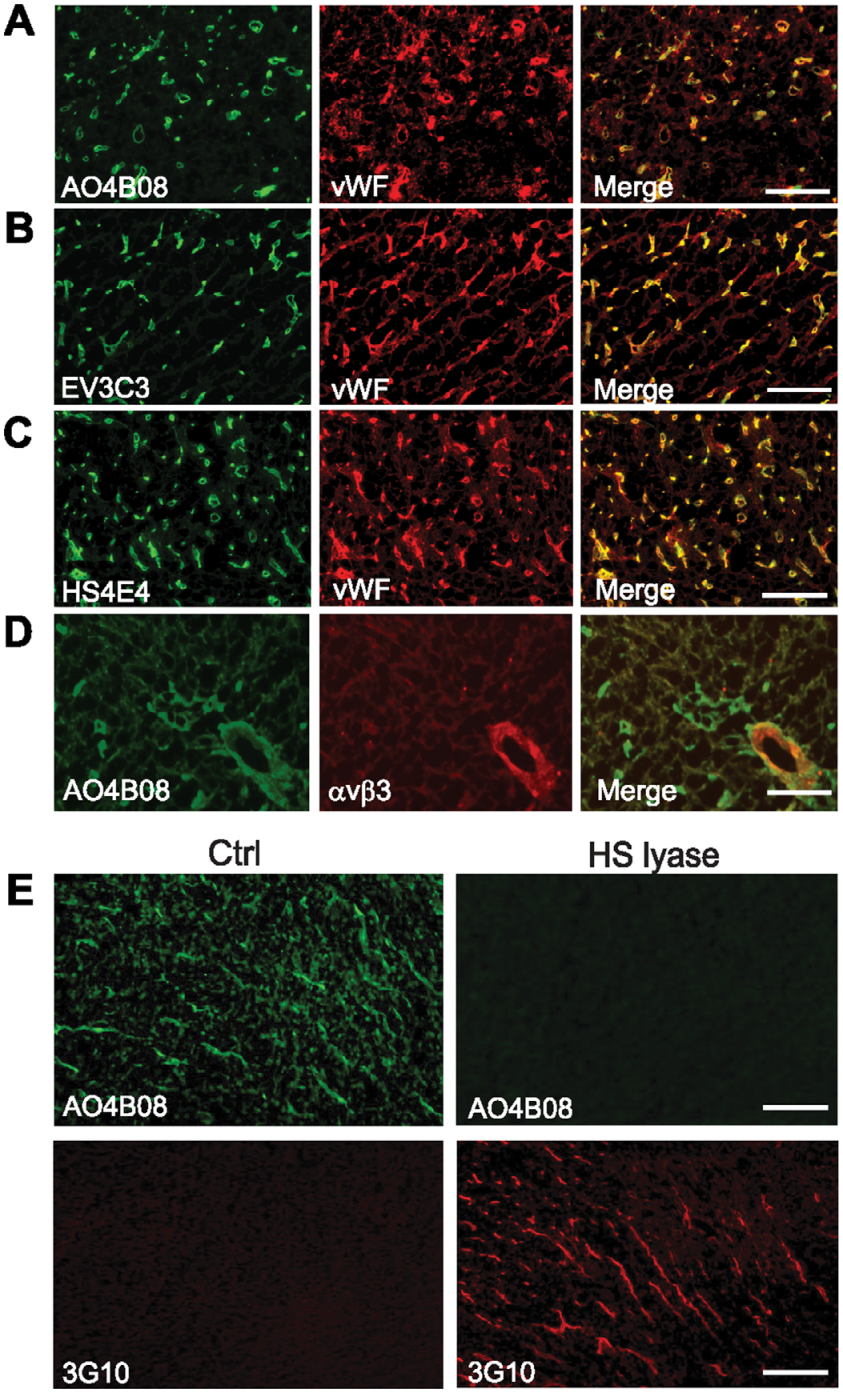
Identification of αHS that preferentially recognize HS epitopes of tumor vasculature. A–D , Patient glioblastoma tumor sections were stained for HS using the αHS antibodies AO4B08 (A and D), EV3C3 (B) or HS4E4 (C) (green) and for vWF (A–C, endothelial marker; red) or integrin αvβ3 (D, marker of activated endothelium; red), and analyzed by fluorescence microscopy. Strong co-association between HS epitopes and tumor vasculature is indicated by merged images (A–D, right panels; yellow). Images shown are representative of three different tumors and at least five sections per tumor, and were obtained at 20× magnification. Scale bar, 50 µM. **E**, Upper panels: Specificity of αHS was assessed by AO4B08 staining of U-87 MG glioblastoma xenograft tumor sections following either no treatment (Ctrl) or pre-treatment with Heparinase III lyase (HS lyase) to digest HS. Lower panels: 3G10 antibody was used to detect HS stubs of HSPGs that remain after HS lyase digestion. Expecedly, 3G10 staining was not detectable in untreated tumor sections with intact HS (Ctrl, left panel); however, 3G10 staining of HS lyase treated specimens (right panel) shows a vessel like pattern that largely resembles the AO4B08 staining (upper left panel). Scale bar, 20 µM.

We initially sought to identify αHS clones with known epitope specificities (**Table 1**) [26] that may recognize the vascular niche of patient glioblastoma tumors. This tumor type was chosen based on its well-known phenotypic characteristics of hypoxia-induced angiogenesis manifested by profound EC hyperplasia [27]. As shown in **Fig. 1A–D**, three separate αHS clones (AO4B08, EV3C3, and HS4E4) preferentially stained for HS epitopes localized to the tumor vasculature, *i.e.* αHS showed strong co-localization with the EC markers von Willebrand factor (vWF) and integrin αvβ3, the latter being a known marker of activated endothelium [28]. HS epitopes recognized by the same set of αHS antibodies were found to be expressed also in primary human ECs (HUVECs) (**Fig. S1A–C**). The specificity of αHS for HS *in vitro* was shown by markedly reduced binding to ECs following treatment with heparanase lyase that enzymatically degrades HS (**Fig. S1D**). The specificity of αHS for HS was also true *in vivo* in glioblastoma xenograft tumor sections, as heparanase treatment resulted in a complete loss of αHS staining (**Fig. 1E**, right upper panel). We next performed stainings with an antibody (3G10) that is known to specifically recognize HSPG core protein with a remaining HS stub following treatment with heparanase, but that does not recognize either free HS chains or intact HSPG [29]. Untreated tumor sections showed no staining with 3G10 (**Fig. 1E**, lower left panel), whereas tumor sections treated with heparanase displayed substantial positivity for the 3G10 epitope predominantly localized to vessel-like structures that closely resembled the αHS staining pattern (cf. **Fig. 1E**, lower right panel and upper left panel). Together, these studies identify several αHS clones that recognize HS epitopes associated with intact HSPGs abundant in the vascular niche of glioblastoma tumors.

**Table 1:** αHS used in this study and their epitope specificities.*

Clone	VH	DP	CDR3	GAG	Modifications involved inbinding	Modifications that may inhibit binding
AO4B08	3	47	SLRMNGWRAHQ	HS	NS, IdoA, 2-OS, 6-OS	
HS4E4	3	38	HAPLRNTRTNT	HS	NAc, NS, IdoA	2-OS, 6-OS
EV3C3	3	42	GYRPRF	HS	NS, IdoA, 2-OS	6-OS

CDR3, amino acid sequence of the complementarity determining region 3 (a major determinant in antigen recognition); DP, gene number; GAG: class of glycosaminoglycan with which the antibody reacts; IdoA, iduronic acid; NAc, N-acetylation; VH, heavy chain germ line family.

* Adapted from reference [26].

### Functional Effects of αHS in Primary Human ECs

**Figure 2:**
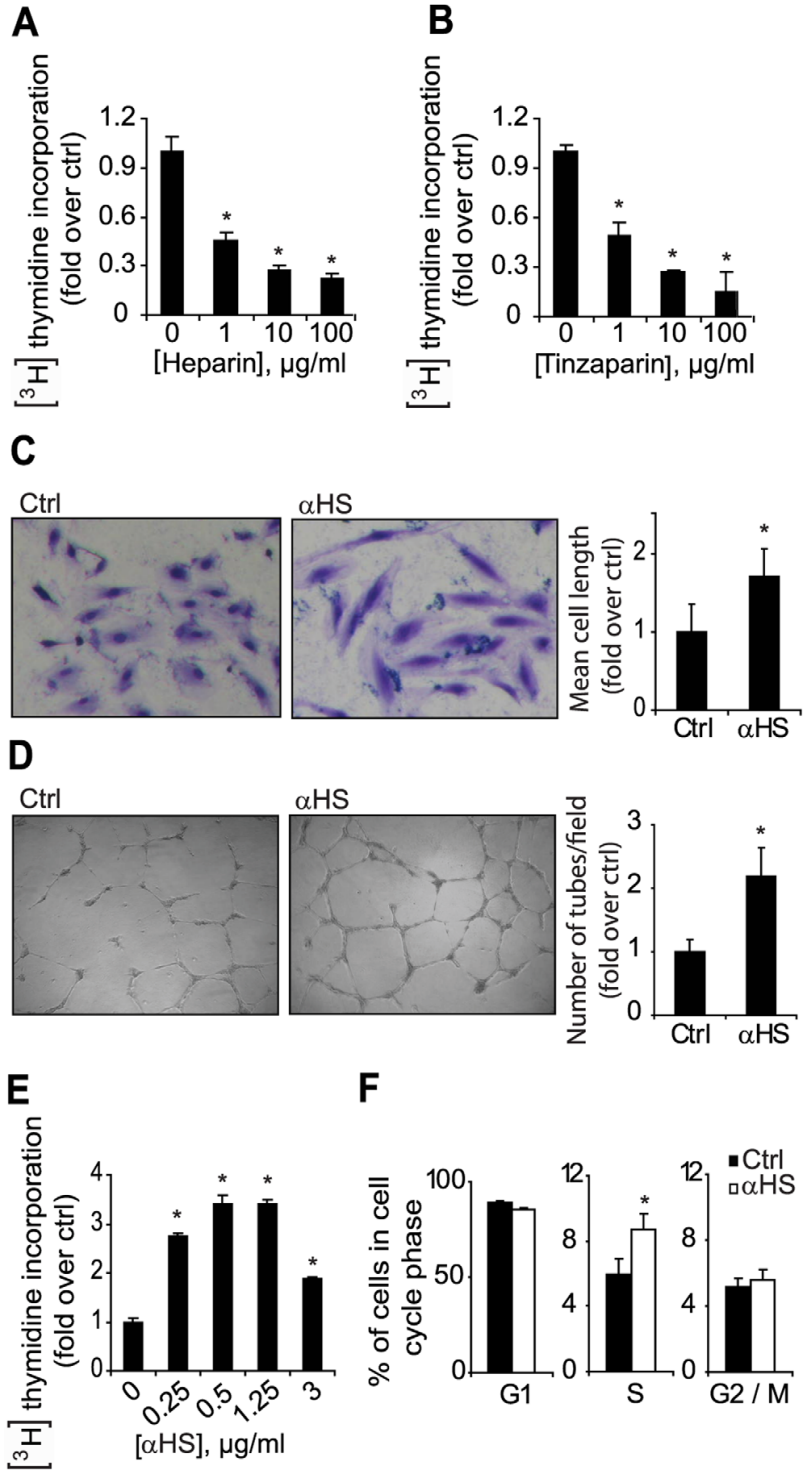
Potent pro-angiogenic effects of αHS in primary human ECs. A–B , HUVECs were grown in serum free medium in the presence of the indicated concentrations of either heparin (A) or the low molecular weight heparin, Tinzaparin (B). Cell proliferation was determined by measurement of incorporated [^3^H]thymidine over a period of 48 h. **C**, HUVECs were grown in serum free medium in the absence (Ctrl) or presence of AO4B08 (αHS, 3 µg/ml) for 72 h. Cell morphology was visualized by crystal violet staining, and captured using an inverted light microscope equipped with a digital camera. Left and mid panels show representative pictures of an elongated EC phenotype in αHS-treated as compared with control cells. Right panel shows quantitative analysis of the length of individual cells in three separate microscopic fields per well (n = 4), using Image J software. **D**, HUVECs were cultured on matrigel, *i.e.* a tumor-derived extracellular matrix, in serum free medium in the absence (Ctrl) or presence of AO4B08 (αHS; 3 µg/ml) and were allowed to form tubes for approximately 20 h. Left and mid panels show representative pictures of increased tube formation in αHS-treated as compared with control cells from at least three independent experiments. Right panel shows quantitative analysis of the number of intact tubes from three microscopic fields per well (n = 4) using Image J software. **E and F**, αHS stimulates EC proliferation: HUVECs were grown in serum free medium in the absence or presence of AO4B08 (αHS) at the indicated concentrations and assessed for proliferation by [^3^H]thymidine incorporation over a period of 3 h. **F**, HUVECs were subjected to cell cycle phase analysis after 6 h without (Ctrl) or with AO4B08 (αHS) treatment. Fraction of cells in the respective cell cycle phase was determined by flow cytometry following nuclear staining with propidium iodide. Data are presented as the average ± S.D. * Statistically different from untreated Ctrl, P<0.05.

Based on the hypothesis that HSPGs are suitable targets for anti-angiogenic treatment [3]–[10], [13]–[18] we next performed a series of functional studies with primary human ECs. Consistent with a role of HSPGs in EC function, the HS mimetic heparin markedly reduced EC proliferation in a dose-dependent manner (**Fig. 2A**). The same was true for the low molecular weight heparin compound tinzaparin (**Fig. 2B**), which is a widely used anticoagulant and that is currently under investigation for its tumor inhibiting effects in lung cancer patients (http://clinicaltrials.gov/ct2/show/NCT00475098?term=tinzaparincancer&rank=7).

A significant drawback with heparins is their unspecific mechanism of action and potentially serious adverse effects. We thus decided to explore the possibility of antibody-based targeting of HS as a more specific and rational anti-angiogenic strategy, initially studying one of the previously selected αHS, *i.e.* AO4B08, on EC function. AO4B08 treated ECs displayed an elongated morphology with an approximately 75% increase of mean cell-length as compared with untreated cells (**Fig. 2C**). EC elongation is known to be associated with a tube forming and sprouting EC phenotype [30]. Accordingly, AO4B08 was found to promote tube formation >2-fold as compared with control cells (**Fig. 2D**). Moreover, αHS was found to substantially increase EC proliferation in a dose-dependent manner with a maximal stimulation of approximately 3.5-fold as compared with control (**Fig. 2E**). Notably, AO4B08 mediated stimulation of EC proliferation was shown already at 3 h of treatment, suggesting a relatively rapid response. The effect of AO4B08 on EC proliferation was sustained after 24 h of AO4B08 treatment (**Fig. S2**). Stimulation of EC proliferation by AO4B08 was further supported by an increased fraction of cells in S-phase, and a corresponding reduction of G1-phase in AO4B08 treated as compared with control cells (**Fig. 2F**).

### αHS Protects Primary Human ECs from Hypoxia and Starvation Associated Cell-death

**Figure 3:**
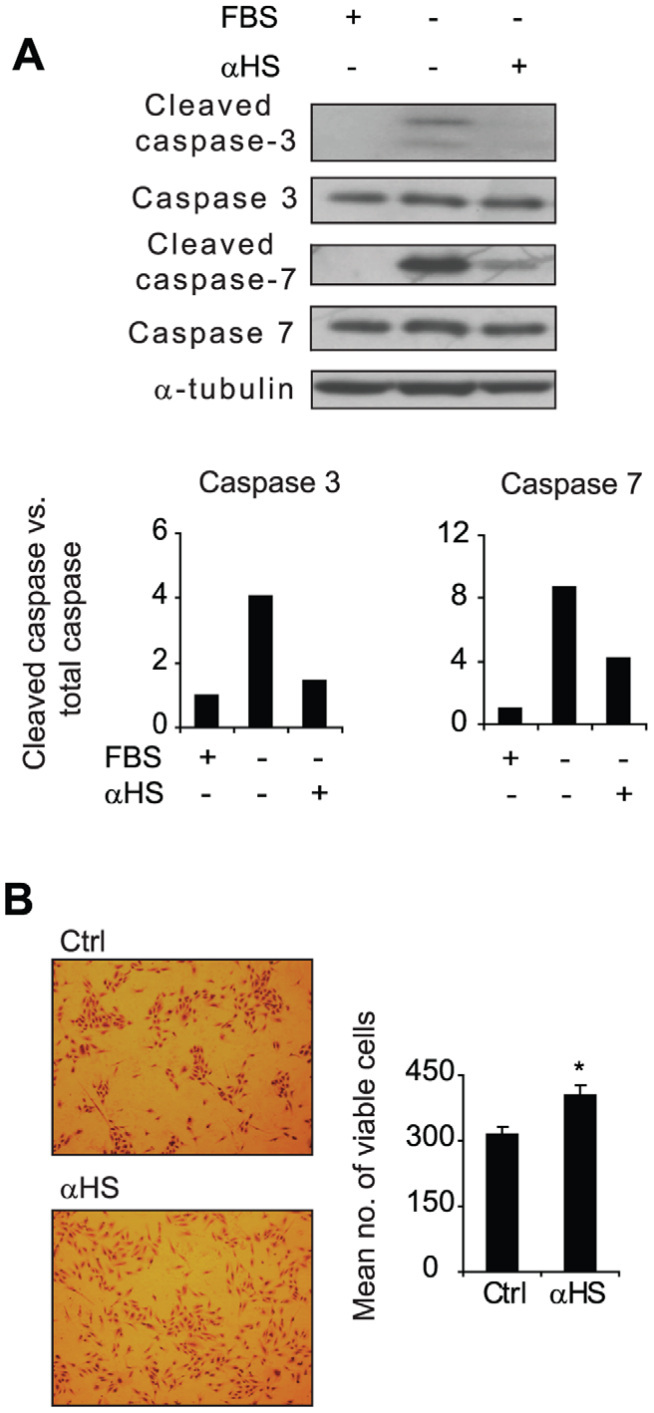
αHS counter-acts hypoxia and starvation induced cell-death in ECs. A , HUVECs were grown for 72 h in full medium (10% FBS), serum free medium, or in serum free medium supplemented with AO4B08 (αHS; 3 µg/ml), as indicated, under hypoxic conditions (1% O_2_). Cell lysates were analyzed for total and cleaved caspase-3 and 7 as well as α-tubulin by immunoblotting. Upper panel shows representative immunoblots from three independent experiments. Lower panels show quantification of cleaved caspase/total caspase ratios from a representative experiment. **B**, HUVECs were grown in serum free medium in the absence (Ctrl) or presence of AO4B08 (αHS; 3 µg/ml) under hypoxic conditions for 72 h, and then stained with crystal violet. Left panel shows representative images of stained cells. Right panels shows quantitative analysis of viable cells. Data are presented as the average±S.D. *Statistically different from untreated Ctrl, P<0.05.

As hypoxia and nutrient deficiency are characteristic features of the tumor microenvironment [31], and as hypoxia has been shown to modulate the cell-surface HS composition of ECs into an increased growth factor binding capacity [32], we reasoned that antibody-mediated blockage of HS may sensitize ECs in the context of hypoxia and starvation. To this end, ECs were cultured at hypoxia (1% O_2_) in the presence or the absence of AO4B08, and assessed for apoptosis by analysis of cleaved caspases. Serum starvation of hypoxic ECs induced caspase 3 and 7 activation in ECs and, interestingly, caspase cleavage was efficiently counter-acted by AO4B08 treatment (**Fig. 3A**). In further support of these data, AO4B08 was shown to significantly increase the number of viable ECs at hypoxic and serum-deficient conditions (**Fig. 3B**). Altogether, the above data suggest that an αHS shown to recognize tumor vasculature-resident HS structures may exert pro-angiogenic effects in ECs.

### αHS Mediated Cell Stimulation is Specific for HS but is not HS Epitope or Cell Specific

**Figure 4:**
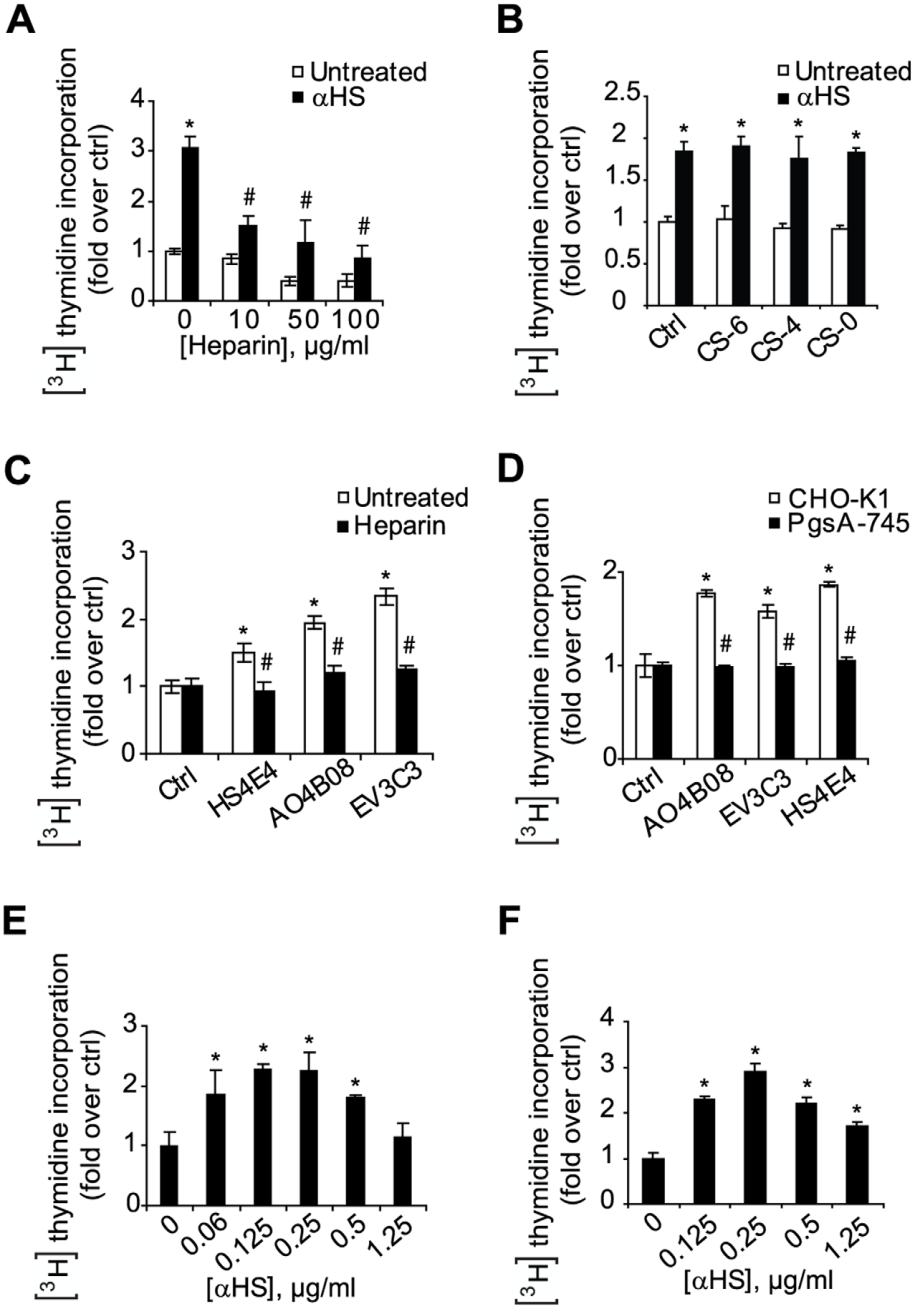
αHS stimulates the proliferation of ECs and transformed cells in an HSPG-dependent manner. **A**, HUVECs were grown in serum free medium in the absence (white bars) or the presence of AO4B08 (black bars; 1.25 µg/ml) and with the indicated concentration of heparin for 3 h and assessed for cell proliferation by the [^3^H]thymidine incorporation assay. *Statistically different from untreated control, P<0.05; # Statistically different from AO4B08-treated, P<0.05. Control cells were incubated without antibody. **B**, HUVECs were grown in serum free medium in the absence (white bars) or the presence of AO4B08 (black bars; 1.25 µg/ml) without (Ctrl) or with CS-6, CS4, or CS-0 (10 µg/ml), as indicated. Cell proliferation was determined by the [^3^H]thymidine incorporation assay. *Statistically different from untreated control, P<0.05. **C**, HUVECs were grown in serum free medium in the absence (white bars) or the presence of heparin (black bars; 10 µg/ml) without or with the different αHS antibody clones (1.25 µg/ml), as indicated. Cell proliferation was determined by the [^3^H]thymidine incorporation assay. *Statistically different from untreated control, P<0.05; # Statistically different from αHS-treated, P<0.05. **D**, Wild type (CHO-K1) cells and PG- deficient (PgsA-745) CHO cells were grown in serum free medium in the absence or the presence of different αHS antibody clones (1.25 µg/ml), as indicated. Cell proliferation was determined by the [^3^H]thymidine incorporation assay. *Statistically different from untreated control, P<0.05; # Statistically different from αHS-treated CHO-K1 cells, P<0.05. **E** **and F**, Human umbilical artery ECs (HUAECs; E) and U-87 MG glioblastoma cells (F) were grown in serum free medium in the presence of AO4B08 (αHS) at the indicated concentrations and proliferation was determined by the [^3^H]thymidine incorporation assay. *Statistically different from untreated control, P<0.05.

We next investigated the specificity of the functional effects of αHS. AO4B08 stimulation of EC proliferation appeared strictly dependent on HS binding as indicated by efficient reversal by heparin in a dose-dependent manner (**Fig. 4A**). Already at the lowest heparin concentration used (10 µg/ml), approximately 75% of AO4B08-dependent proliferation was inhibited, and at 100 µg/ml the effect was completely abrogated (**Fig. 4A**). Importantly, we further found that additional αHS clones shown to recognize glioblastoma tumor vasculature, HS4E4 and EV3C3 (**Fig. 1**), also stimulated EC proliferation, and that this effect was efficiently counter-acted by heparin (**Fig. 4C**). It should be noted that in the proliferation experiments shown in **Fig. 2A**, cells were treated with heparin for 48 h, whereas in the αHS reversal experiments cells were incubated with heparin only for 3 h (**Fig. 4A and C**). Importantly, various variants of CS, *i.e.* a glycosaminoglycan closely related to heparin, was unable to reverse the stimulatory effect of αHS (**Fig. 4B**). Moreover, under these conditions, heparin (10 µg/ml) efficiently inhibited αHS cell-surface binding (**Fig. S3B**). Although, these results indicate that αHS-mediated effects on EC proliferation are HS specific but not restricted to a specific HS epitope, the stimulatory effect was most pronounced for EV3C3 followed by AO4B08 and HS4E4 (**Fig. 4C**). These results seemed to correlate with the cell-surface abundance of the respective αHS epitope, *i.e.* ECs preferentially bound αHS in the order EV3C3>AO4B08>HS4E4 (**Fig. S3A**). Further, the effect of the various αHS clones appeared not to be directly linked to the cellular distribution of their respective epitope, as AO4B08 and HS4E4 stainings displayed a similar pattern along the cell periphery, whereas EV3C3 showed a more diffuse staining with increased density towards the perinuclear region (**Fig. S1A–C**).

The specificity of αHS and the dependence on HSPG for their stimulatory effects were next investigated using wild-type (CHO-K1) and mutant (pgsA-745) CHO cells, genetically deficient in xylosyl transferase (XT). XT catalyses the first step in HSPG assembly, *i.e*. the linkage of xylose to specific serine residues in the PG core protein. PgsA cells express approximately 5% HSPG as compared with wild-type cells [33]. Treatment of CHO-K1 cells with AO4B08, EV3C3 as well as HS4E4 resulted in an almost 2-fold increase in proliferation, showing that the stimulatory effects of αHS were not restricted to HUVECs (**Fig. 4D**). This notion was reinforced by the finding that αHS induced the proliferation of human umbilical artery ECs (HUAEC), *i.e.* another source of primary human ECs, as well as the human glioblastoma cell-line U-87 MG in a dose-dependent manner and to the same extent as in HUVECs (**Fig. 4E and F**). Importantly, HSPG-deficient mutant cells were unresponsive to all of the studied αHS (**Fig. 4D**), providing genetic evidence that αHS-mediated activation of cell proliferation requires unperturbed HS function.

### αHS Dependent Stimulation of Proliferation Involves p38 MAPK Signaling Activation

**Figure 5:**
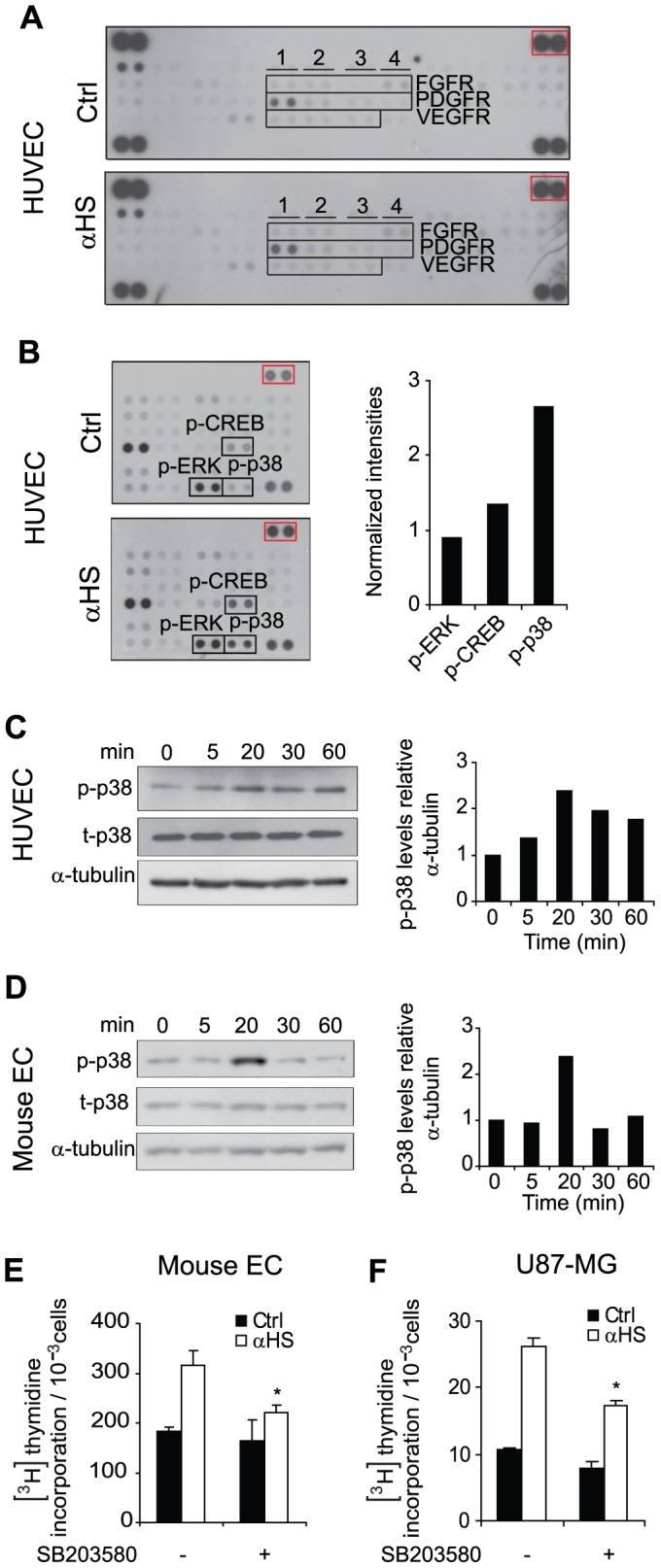
αHS-induced proliferation of ECs and transformed cells depends on p38 MAPK signalling activation. A, HUVECs were incubated in serum free medium with or without αHS (AO4B08; 1.5 µg/ml) for 5, 15, 45, and 60 min, and phosphorylated receptor tyrosine kinase proteins were determined on pooled cell lysates by human anti-phospho receptor tyrosine kinase antibody array analysis as described under *Methods*. Shown are representative immunoblots from two independent experiments. Indicated areas show FGFR, column 1: FGFR1; 2: FGFR2; 3: FGFR3; 4: FGFR4. PDGFR, column 1: PDGFRα; 2: PDGFRβ; 3: SCFR; 4: Flt3. VEGFR, column 1: VEGFR1; 2: VEGFR2; 3: VEGFR3. **B**, HUVECs were incubated in serum free medium with or without αHS (EV3C3; 1.5 µg/ml) for 5, 15, 45, and 60 min, and phosphorylated kinase proteins were determined on pooled cell lysates by human anti-phospho kinase antibody array analysis as described under *Methods*. Left panels show representative immunoblots from three independent experiments. Right panel shows quantification of p-p38 MAPK, p-CREB, and p-ERK1/2 levels at the various conditions, presented as relative intensities *vs.* array reference (red box). **C**, HUVECs were either untreated (Ctrl) or incubated with αHS (AO4B08;1.25 µg/ml) for the indicated time periods followed by immunoblotting for phospho-p38 MAPK, total p38 MAPK and α-tubulin. Left panel shows representative immunoblots of three separate experiments. Right panel shows quantification of relative phospho-p38 MAPK levels (P-p38/α-tubulin) from a representative experiment. **D**, Same experiment as in (C) was performed with mouse lung microvascular ECs, showing comparable induction of p38 MAPK phosphorylation by αHS as in HUVECs. **E–F**, αHS-induced proliferation depends on p38 MAPK. Proliferation was determined by [^3^H]thymidine incorporation in mouse ECs (**E**) and U-87 MG glioblastoma cells (**F**) following treatment with the p38 MAPK inhibitor SB203580 (1 µM) and/or αHS (AO4B08; 1.25 µg/ml) for 3 h, as indicated. Basal cell proliferation was not significantly affected by p38 MAPK inhibition whereas αHS induced proliferation was almost completely reversed. * Statistically different from untreated control, P<0.05; # Statistically different from αHS-treated, P<0.05.

We next wanted to elucidate whether αHS induced cell proliferation involved a specific signaling pathway. Using a phosphorylation-specific receptor tyrosine kinase array, we found that αHS treatment did not activate receptors of known HS-binding growth factors, *i.e.* FGFs, PDGFs, and VEGFs, as compared to untreated ECs (**Fig. 5A**). These results suggest that the mechanism of αHS-mediated stimulation of ECs is not due a direct mimicking effect of HS-binding ligands. Interestingly, using a phosphorylation-specific kinase array, αHS treatment was found to significantly induce phosphorylation of the p38 MAPK (approximately 2.5-fold) as compared to untreated ECs (**Fig. 5B**). The array data were confirmed by western blotting, showing time-dependent and transient induction of p-p38 MAPK by αHS (**Fig. 5C**). Moreover, phosphorylation of CREB, a known downstream target of p38 MAPK [34], appeared to be increased upon αHS treatment (by approximately 35% as compared with control) (**Fig. 5B**). However, phosphorylation of another MAPK widely implicated in proliferation signaling, extracellular signal regulated kinase 1/2 (ERK1/2), was unaffected by αHS treatment (**Fig. 5B**). The effect of αHS on p38 MAPK signaling activation appeared to be a more general phenomenon, as a similar response was found in microvascular ECs isolated from mouse lung (**Fig. 5D**) [35] as well as in U-87 MG glioblastoma cells (**Fig. S4A**).

Several growth factors, perhaps most importantly pro-angiogenic FGF, VEGF, and PDGF have been found to trigger p38 MAPK activation in certain cell types [36]. We thus performed a series of angiogenesis and tyrosine kinase receptor phosphorylation antibody array experiments to elucidate whether cell stimulatory effects and p38 MAPK activation by αHS may be secondary to the induction of specific growth factors and their respective signaling receptors. However, we were unable to find significant induction by αHS either of these growth factors or their receptors (data not shown). To directly study the functional importance of p38 MAPK signalling in αHS-mediated proliferation, we employed the well-established p38 MAPK inhibitor SB203580 [34], [37]. Expectedly, the inhibitor efficiently counter-acted p38 MAPK activation in HUVECs (**Fig. S4B**), and reduced serum as well as αHS-mediated activation of the p38 MAPK down-stream target CREB (**Fig. S4C)**. SB203580 was shown to inhibit basal p-CREB (**Fig. S4C**) and proliferation in non-stimulated HUVECs (data not shown), but not in mouse ECs and U-87 MG glioblastoma cells (**Fig. 5E and F**), *i.e.* further studies were performed in the latter cell types. Interestingly, αHS-induced proliferation of mouse ECs (**Fig. 5E**) as well as glioblastoma cells (**Fig. 5F**) was efficiently attenuated by p38 MAPK inhibition. Together, these data indicate that binding of αHS to cell-surface HSPGs of ECs as well as glioblastoma cells triggers a p38 MAPK-dependent signaling pathway resulting in increased proliferation.

## Discussion

Antibody based tumor vascular targeting for the treatment of cancer is based on the fact that tumors require angiogenesis in order to expand and metastasize, and that ECs face the lumen of blood vessels and thus are relatively accessible for systemic therapy. The rationale of HSPG-directed anti-tumor therapy is the possibility of simultaneous multi-targeting of some key pro-angiogenic proteins, most importantly VEGF-A, FGFs, HB-EGF, PDGF, and IL-8, which may promote tumor angiogenesis in a partly redundant manner with implications for treatment resistance. In the present study, we have identified several αHS clones that recognize HS epitopes abundantly expressed in the vasculature of malignant glioma tumors. Unexpectedly, we find that these αHS stimulate several aspects of EC function, *i.e.* differentiation, tube formation, proliferation and resistance to hypoxia and starvation dependent cell-death. We provide evidence that these effects are specifically dependent on αHS binding, and are not related to unspecific interactions. Importantly, the stimulatory effects appeared to involve several HS epitopes, as AO4B08, EV3C3 as well as HS4E4 all displayed stimulatory activity, which appeared to be directly correlated with their respective cell-surface abundance in ECs. Furthermore, αHS-induced stimulation was found in three different types of primary ECs, *i.e.* HUVECs, HUAECs, and mouse lung microvascular ECs, as well as in transformed CHO cells and human U-87 MG glioblastoma cells, suggesting that our findings have more general relevance.

Previous studies lend support to the notion of HSPG as a relevant target of cancer therapy [7]–[18]. These strategies have mainly been focused on heparin, its derivatives, and other polyanionic compounds, such as suramin [18]. Other strategies to interfere with HSPG function include false substrates for HS biosynthesis, *i.e.* xylosides [38], inhibition of HS-degrading enzymes [39], and genetic manipulation of the HS biosynthetic machinery [40]. The demonstrated inhibitory effects of these compounds can mainly be explained by direct competitive blockage of ligand-HSPG interactions thereby attenuating down-stream receptor signaling activation (polyanionic compounds), or by modulating the biological availability of HSPGs for ligand binding (xylosides, enzymes, genetic strategies). Interestingly, our data indicate that the dominant effect of antibody binding to cellular HSPGs is to directly promote cell activation, rather than to block HS ligand-dependent cell activation. On a mechanistic level, binding of αHS to HSPGs of ECs as well as glioblastoma cells was found to trigger p38 MAPK-dependent signaling, and unperturbed p38 MAPK function was required for αHS-mediated cell stimulation. In this context, it is notable that other HS-binding ligands have been shown to stimulate ECs through the p38 MAPK pathway [41]. This intracellular kinase was initially identified as a major pro-inflammatory mediator and was later found to be involved in multiple cellular processes and in diseases including cancer [36]. In fact, p38 MAPK has been implicated as a suppressor of malignant processes, *e.g.* oncogene-mediated cell senescence and contact inhibition. Other studies, however, have shown pro-migratory and invasive effects of p38 MAPK activation in cancer cells and stromal cells [36]. Like other MAPKs, the functions of p38 are dependent on its down-stream kinases, including serine/threonine kinases such as p38-regulated/activated kinase (PRAK) and MAPK-activated kinase-2, and many different up-stream kinases, such as MKK3/6, MKK4, as well as MAPKK independent signals. Cell specific as well as subcellular localization specific effects add to the complexity of p38 function. In the context of tumor angiogenesis it is of particular interest that p38 was recently shown to activate tumor ECs through PRAK [42]. Future studies will have to clarify whether PRAK and what alternative p38 down-stream signaling mechanisms are involved in αHS-mediated pro-angiogenic effects. Moreover, our studies were limited to the kinases detected by the antibody array, *i.e.* other kinases not included in the array could be activated by αHS in addition to p38.

The dominating cell-surface HSPGs are the syndecan and glypican families that have both been implicated in the regulation of growth factor signaling and angiogenesis [6], [7]. The syndecans are transmembrane HSPGs that have a direct role in cell signaling events, *e.g.* the cytoplasmic domain of syndecan-4 contains a central region that binds phosphatidylinositol 4 5-bisphosphate, which allows oligomerization of syndecan with subsequent binding and activation of protein kinase C-alpha [43]–[44]. Syndecan-4 has also been shown to promote the formation of actin stress fibers and focal adhesions in conjunction with the integrins [45]. Interestingly, glypican-1 was highly expressed in glioma vasculature, while absent in normal brain ECs. Further, induced over-expression of glypican-1 in normal ECs resulted in increased mitogenesis [46], [47]. The glypicans are linked to the cell surface via a lipid-anchor, which localizes this type of HSPG to cholesterol-rich membrane raft/caveolae domains [6]. Notably, recent studies point at an important role of membrane-raft associated endocytosis in signaling regulation [48], and previous studies from our group have shown that HSPGs are endocytosed mainly through this pathway [49]. It is thus tempting to speculate that αHS binding induces clustering of glypicans at the cell-surface, which results in raft assembly, endocytosis, p38 MAPK signaling activation and the observed functional effects. However, our data are not in favor of this idea, as HS4E4, EV3C3 as well as AO4B08 showed cell stimulatory activities, whereas only the latter has been shown to exert internalizing capacity [50].

We conclude that several αHS clones that recognize HS epitopes abundant in the tumor vasculature activate ECs as well as transformed cells *in vitro*, which irrespective of the underlying mechanism, may have important implications for the development of antibody-based targeting of HSPGs in cancer. Our results motivate further *in vivo* studies to determine the overall effects of αHS on tumor development, and to investigate potential adverse effects related to antibody-mediated HS blockage at the systemic level.

## Methods

### Ethics Statement

Mice were kept under pathogen-free conditions in the isolation facility at the Biomedical Center, Lund University, in accordance with the Swedish guidelines for humane treatment of laboratory animals. The experimental setup was approved by the ethical committee for animal research in Malmö/Lund, Sweden. Patient glioblastoma samples were obtained from the Neurosurgery Department, Lund University. Ethical permission for the present investigation was obtained from the Lund University Regional Ethics Board, whereby informed consent was deemed not to be required as data were analyzed anonymously.

### Materials

ScFv αHS antibodies raised against HS isolated from bovine kidney (HS4E4), skeletal muscle from mouse (AO4B08), and human lung (EV3C3) were used (**Table 1**) [26]. Mouse anti-vsv antibody, clone p5d4, heparinase I (EC 4.2.2.7) and -III (EC 4.2.2.8) were from Sigma. Anti-integrin αvβ3, anti-von Willebrand factor, and anti-α-tubulin antibodies were from Abcam, and Alexa fluor-conjugated secondary antibodies and Phalloidin-TRITC from Invitrogen. Anti-phospho-p38 MAPK, anti-p38 MAPK, and anti-caspase-3 and 7 antibodies were from Cell signaling. Human phospho-kinase and phospho-receptor tyrosine kinase antibody array (#ARY003 and #ARY001) kits were from R&D. P38 MAPK inhibitor (SB203580) was from Tocris, full-length heparin and CS preparations from Sigma, and the low-molecular weight heparin Tinzaparin from Leo Pharma. [^3^H]thymidine was from Amersham, and growth factor reduced Matrigel from BD. Fine grade chemicals and cell media supplements were from Sigma.

### Animal Xenograft Glioblastoma Tumors

Eight-week-old female NOD/SCID mice were inoculated via s.c. injection on the dorsal region with U-87 MG cells (2.5×106 in 150 µl PBS). After approximately 4 weeks of tumor growth, animals were anesthetized with isoflurane, tumors were excised, embedded in paraffin or OCT, and stored for further analysis.

### Cell Culture

Human umbilical vein ECs (HUVECs) and human umbilical artery ECs (HUAECs) were from Lonza. CHO-K1, CHO pgsA-745, and U-87 MG glioblastoma cells were from the American Type Culture Collection, and microvascular mouse ECs were established as previously described [35]. ECs were cultured in Endothelial basal medium (EBM), CHO cells in F12K medium and U-87 MG cells in DMEM. Routine culture was performed in a humidified 5% CO_2_ incubator at 37°C using the respective medium supplemented with 1% l-glutamine, 100 U/ml penicillin and 100 µg/ml streptomycin, 10% heat inactivated fetal bovine serum (FBS) for ECs, and 10% FBS for CHO cells and U-87 MG cells. Unless stated otherwise, experiments were performed in medium without serum. HUVECs and HUAECs were used between passage two to six and ECs were cultured on gelatinized cell culture plastics. For hypoxia experiments, cells were incubated in a humidified InVivo_2_ Hypoxia Workstation 400 (Ruskin Technology) set at 5% CO_2_, 94% N_2_ and 37°C.

### Immunofluorescence Microscopy of Glioblastoma Tumors

Snap frozen, human glioblastoma tumor specimens were cryo sectioned at a thickness of 5 µm. Tumor sections were rehydrated in PBS containing 0.05% tween-20 (PBST) for 10 min and blocked with PBST containing 2% (w/v) BSA (BSA/PBST) for 1 h followed by incubation with c-myc tagged αHS antibody (AO4B08, EV3C3 or HS4E4; 10 µg/ml) together with anti-integrin αvβ3 antibody or anti-von Willebrand factor at 4°C overnight. Sections were washed extensively in PBST, followed by incubation with anti-c-myc antibody for 45 min at room temperature. Sections were washed and incubated with goat-anti mouse Alexa Fluor 488-conjugated antibody and goat-anti rabbit Alexa Fluor 546-conjugated antibody for 1 h at room temperature. Control sections were stained with anti-c-myc antibody and Alexa Fluor-conjugated antibodies in parallel. Sections were mounted with Flourescent Mounting Medium (Dako) and were analyzed using a Zeiss Axio Observer.Z1 HBO 100 fluorescence microscope.

### HS Lyase Digestion

For HS digestion experiments, U-87 MG mouse xenograft cryo sections were incubated in digestion buffer (DMEM supplemented with (25 mM Tris, 50 mM CalCl_2_, 50 mM NaAc, pH 8) with or without Heparinase III (16 mIU/ml) overnight at 37°C. Sections were rinsed extensively in PBST, and stained with αHS or 3G10 antibody as described above.

### Confocal Laser Scanning Microscopy

HUVECs were cultured in eight well chamber slides and fixed in 2% (w/v) paraformaldehyde for 5 min on ice. Cells were blocked in PBS/BSA for 1 h followed by αHS-staining as described. Cells were counter-stained for f-actin using Phalloidin-TRITC accordoing the to the instructions by the manufacturer. Cells were analyzed using a Zeiss LSM 710 confocal scanning equipment with a C-Apochromate 63×/1 1.2 korr W objective and analysed with Zen software.

### Cell-surface αHS Binding

For αHS binding, cells were detached with (2×) PBS/0.5 mM EDTA. Cells were then either left untreated or treated with 1.8 mU/ml heparinase III and 0.9 mIU/ml heparinase I for 2 h in digestion buffer (DMEM supplemented with 0.5% (w/v) BSA and 20 mM HEPES-HCl, pH 7.4), enzymes were added again for another 2 h. αHS staining was performed essentially as described for tumor sections; cells were extensively washed with PBS/BSA in between antibody incubations and all incubations were performed in PBS/BSA for 30 min on ice. αHS cell-surface binding was analyzed by flow cytometry on a FACS Calibur instrument integrated with CellQuest software (BD Biosciences). Control cells without primary αHS were included in the binding experiments.

### Cell Proliferation Assay

Proliferation was determined by [^3^H]thymidine incorporation in serum free medium. Cells were cultured in the presence or absence of heparin and tinzaparin for 48 h or αHS for 3 h together with [^3^H]thymidine (12.5 µCi/ml), as described in the respective Figure legend. Proliferation experiments were in some cases performed with αHS and heparin or CS variants at the indicated concentrations in the respective figure legend. In other experiments, cells were pre-treated with the p38 MAPK inhibitor SB203580 (1 µM). Cells were washed extensively with ice cold PBS, followed by incubation with 10% (w/v) trichloric acid for 30 min on ice. Cells were washed and lysed in 0.1 M NaOH, and an aliquot of cell lysates was neutralized with 0.1 M HCl and analyzed for [^3^H]thymidine incorporation by scintillation counting in a Beckman Coulter LS6500 Liquid Scintillation Counter. For cell cycle phase analysis cells were treated with AO4B08 for 6 h, detached with trypsin and washed with PBS/BSA. Cells were pelleted, resuspended in 70% ice-cold ethanol and stored at −20°C until analyzed. Prior to analysis the samples were resuspended in Nonidet-P40 containing 7 mg/ml Propidium Iodide and 0.1 mg/ml RNase followed by 30 min incubation at room temperature. Cell cycle phase was determined based on DNA content, as analyzed by flow cytometry.

### EC Elongation Assay

HUVECs were grown in the absence or presence of 3 µg/ml αHS for 72 h. Cells were stained using crystal violet and captured using a 4× objective (Axiovert 40C, Carl Zeiss inverted microscope) and a digital camera to assess cell morphology. Cell length was measured for individual cells in three microscopic fields per well (n = 4) using Image J software (National Institutes of Health).

### Tube Formation Assay

HUVECs were seeded on growth factor reduced Matrigel at 25 000 cells/well in EBM containing 2% FBS with or without 3 µg/ml αHS. Tubes were allowed to form over night and tubes were captured using a 4× objective (Axiovert 40C, Carl Zeiss inverted microscope) and a digital camera. Intact tubes were counted in three microscopic fields per well (n = 4) using Image J software.

### Survival Assay

Cells were serum-starved and grown under hypoxic conditions (1% O_2_) for 72 h with or without 3 µg/ml αHS. Cells were stained with crystal violet and were captured using a 4× objective (Axiovert 40C, Carl Zeiss inverted microscope) and a digital camera. Surviving cells were counted in three microscopic fields per well (n = 4). For immunoblot analysis, cells were seeded in 6-well dishes and were treated as described. Cell lysate was collected and immunoblotted for cleaved caspases-3 and 7.

### Immunoblot Assay

Cells were washed in ice-cold PBS and lysed in RIPA buffer containing 20 mM Tris-HCl (pH 7.5), 150 mM NaCl, 1 mM Na_2_EDTA, 1 mM EGTA, 1% NP-40, 1% sodium deoxycholate, 2.5 mM sodium pyrophosphate, 1 mM β-glycerophosphate, and Complete Protease Inhibitor cocktail and phosphoSTOP (Roche diagnostics). The lysates were centrifuged at 4500 *g* for 10 min and the pellet was discarded, supernatants were measured for their protein content using the BCA™ protein assay kit (Pierce). Equal amounts of proteins were mixed with 4× NuPage LDS sample buffer (Invitrogen), boiled and separated by electrophoresis in a 4–12% NuPAGE Bis-Tris gel. SeeBlue Plus2 Prestained Standard (Invitrogen) was used as molecular mass standard. The proteins were electroblotted to a PVDF membrane followed by blocking with TTBS containing 5% skim milk for 1 h at room temperature and were incubated with the indicated antibody overnight at 4°C. Primary antibodies were complexed with HRP-conjugated secondary IgG for 1 h at room temperature. Proteins were visualized using ECL Western blotting substrate (Pierce) and the membranes were exposed to X-ray film (Hyperfilm MP, Amersham). Membranes were scanned and densitometry analyses were performed using Image J software.

### Phospho Kinase and Angiogenesis Arrays

HUVECs were untreated or stimulated with 1.5 µg/ml αHS for 5, 15, 45 and 60 min. Cells lysates from the different time-points were prepared according to the kit protocol and pooled for further analysis. Equal amounts of total protein from untreated and αHS-treated cell lysate were analyzed for phosphorylated intracellular and receptor tyrosine kinases using antibody arrays according to the manufactureŕs instructions. For analysis of angiogenesis associated proteins, HUVECs were cultured and treated as above, lysed with RIPA buffer supplemented with Complete protease inhibitor cocktail, and analyzed using angiogenesis antibody array, as recommended by the manufacturer. Array membranes were exposed to X-ray film, scanned and protein intensities were quantified using ImageJ software (NIH).

### Statistical Analysis

Immunostaining and immunoblotting experiments are representative of at least three experiments with similar results. Functional data are presented as the mean±S.D. (n = 3–6). Statistical significance was evaluated with Student’s *t* test using Microsoft Excel; a P-value of <0.05 was considered significant.

## Supporting Information

Figure S1 **AO4B08, EV3C3, and HS4E4 αHS clones show positive staining in human primary ECs. A–C**, Confocal flourescence microscopy of HUVECs shows the expression of HS epitopes recognized by the indicated αHS (green). Cells were counter-stained for f-actin with Phalloidin-TRITC (red). **D,** HUVECs were treated without (Ctrl) or with HS lyase. Cells were surface stained at 4° with vsv-tagged AO4B08 followed by incubation with mouse anti-vsv antibody and Alexa Fluor 488-conjugated rabbit anti-mouse antibody. Cell surface binding of AO4B08 was analysed using flow cytometry as described under the *Methods* section, and shows HS-specific binding of the antibody. *Statistically different from untreated control, P<0.05.
(TIF)

Figure S2 **Early and sustained induction of EC proliferation by αHS.** HUVECs were grown in serum free medium in the absence or presence of the indicated AO4B08 concentrations for 3 h (white bars) or 24 h (black bars), and cell proliferation was assessed by the [^3^H]thymidine incorporation assay. *Statistically different from untreated control, P<0.05
(TIF)

Figure S3 **Differential binding of AO4B08, EV3C3, and HS4E4 αHS clones to ECs. A,** HUVECs were detached by concentrated PBS (x2)/EDTA and surface stained at 4° with AO4B08, EV3C3, or HS4E4 αHS clones (10 µg/ml). αHS cell-surface binding was analysed by flow cytometry. **B**, HUVECs were surface stained at 4° with mouse anti-vsv antibody and Alexa Fluor 488-conjugated rabbit anti-mouse antibody (Ctrl, white area), or with vsv-tagged AO4B08, mouse anti-vsv antibody, and Alexa Fluor 488-conjugated rabbit anti-mouse antibody in the absence (black area) or in the presence of heparin (10 µg/ml; grey area). Cell surface binding was analysed using flow cytometry. Right panel: Data are presented as the average±S.D.
(TIF)

Figure S4 **αHS induces P38 MAPK activation in U-87 MG cells and αHS-mediated signalling is blocked by P38 MAPK inhibition. A**, U-87 MG glioblastoma cells were either untreated (Ctrl) or incubated with αHS (AO4B08; 1.25 µg/ml) for the indicated time periods followed by immunoblotting for phospho-p38 MAPK and α-tubulin. Left panel shows representative immunoblots of three separate experiments. Right panel shows quantification of relative phospho-p38 MAPK levels (p-p38/α-tubulin) from a representative experiment. **B**, Same experiment as in (A) was performed with HUVECs, either untreated (Ctrl) or treated with αHS (AO4B08; 1.25 µg/ml) for the indicated time periods with or without 30 min pre-treatment with p38 MAPK inhibitor SB203580 (2 µM) as indicated. Left panel shows representative immunoblots of three separate experiments. Right panel shows quantification of relative phospho-p38 MAPK levels (p-p38/α-tubulin) from a representative experiment. **C**, HUVECs were left untreated or pre-treated with p38 MAPK inhibitor SB203580 (2 µM) for 30 min, stimulated with FBS (10%) or αHS (AO4B08; 1.25 µg/ml), followed by immunoblotting for phospho-CREB, total CREB, and α-tubulin. Left panel shows representative immunoblots of three separate experiments. Right panel shows quantification of relative phospho-CREB levels (p-CREB/α-tubulin) from a representative experiment.
(TIF)
