# Supplementary figures and images for: ScFv Anti-Heparan Sulfate Antibodies Unexpectedly Activate Endothelial and Cancer Cells through p38 MAPK: Implications for Antibody-Based Targeting of Heparan Sulfate Proteoglycans in Cancer
Source: PLoS One. 2012 Nov 9;7(11):e49092. doi: 10.1371/journal.pone.0049092 (PMC3494658; doi:10.1371/journal.pone.0049092)

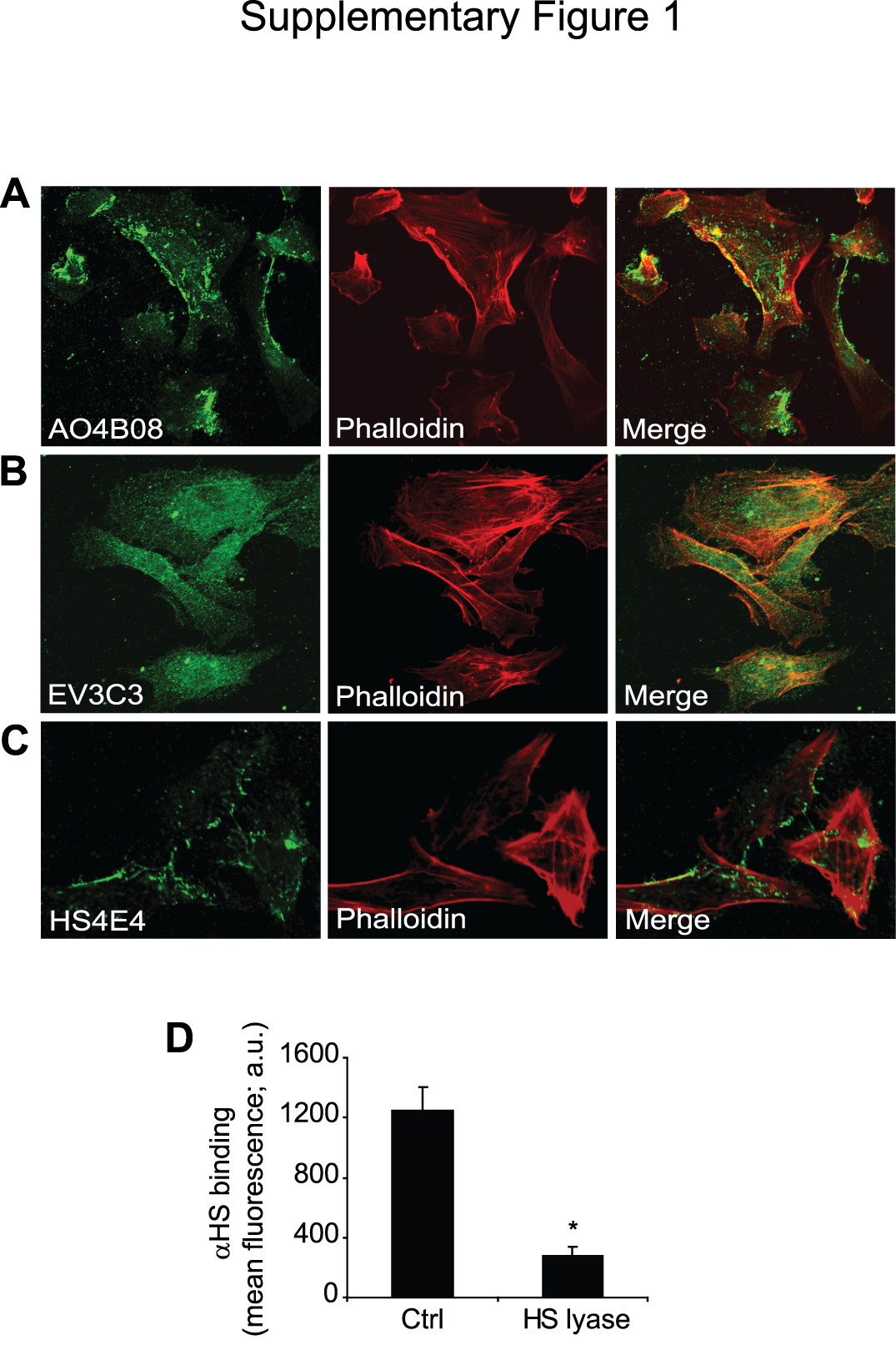

Supplement: Figure S1 — AO4B08, EV3C3, and HS4E4 αHS clones show positive staining in human primary ECs. A–C, Confocal flourescence microscopy of HUVECs shows the expression of HS epitopes recognized by the indicated αHS (green). Cells were counter-stained for f-actin with Phalloidin-TRITC (red). D, HUVECs were treated without (Ctrl) or with HS lyase. Cells were surface stained at 4° with vsv-tagged AO4B08 followed by incubation with mouse anti-vsv antibody and Alexa Fluor 488-conjugated rabbit anti-mouse antibody. Cell surface binding of AO4B08 was analysed using flow cytometry as described under the Methods section, and shows HS-specific binding of the antibody. *Statistically different from untreated control, P<0.05. (TIF) [file pone.0049092.s001.tif]

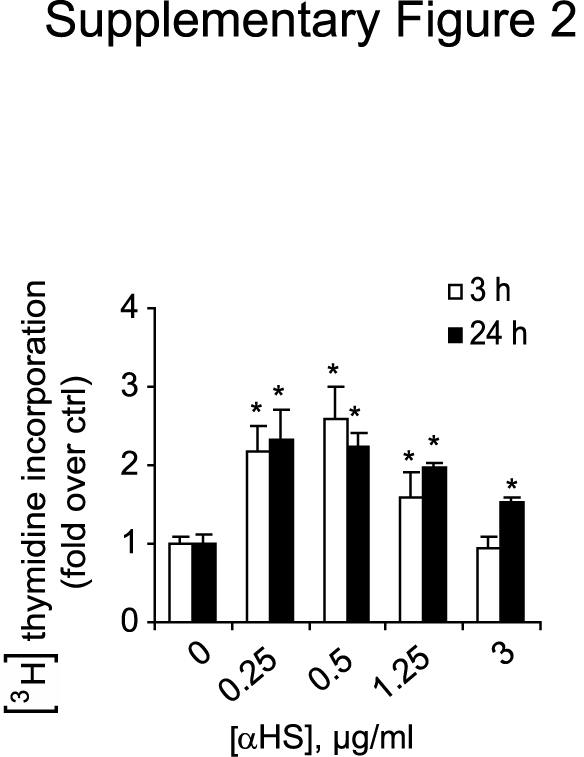

Supplement: Figure S2 — Early and sustained induction of EC proliferation by αHS. HUVECs were grown in serum free medium in the absence or presence of the indicated AO4B08 concentrations for 3 h (white bars) or 24 h (black bars), and cell proliferation was assessed by the [3H]thymidine incorporation assay. *Statistically different from untreated control, P<0.05 (TIF) [file pone.0049092.s002.tif]

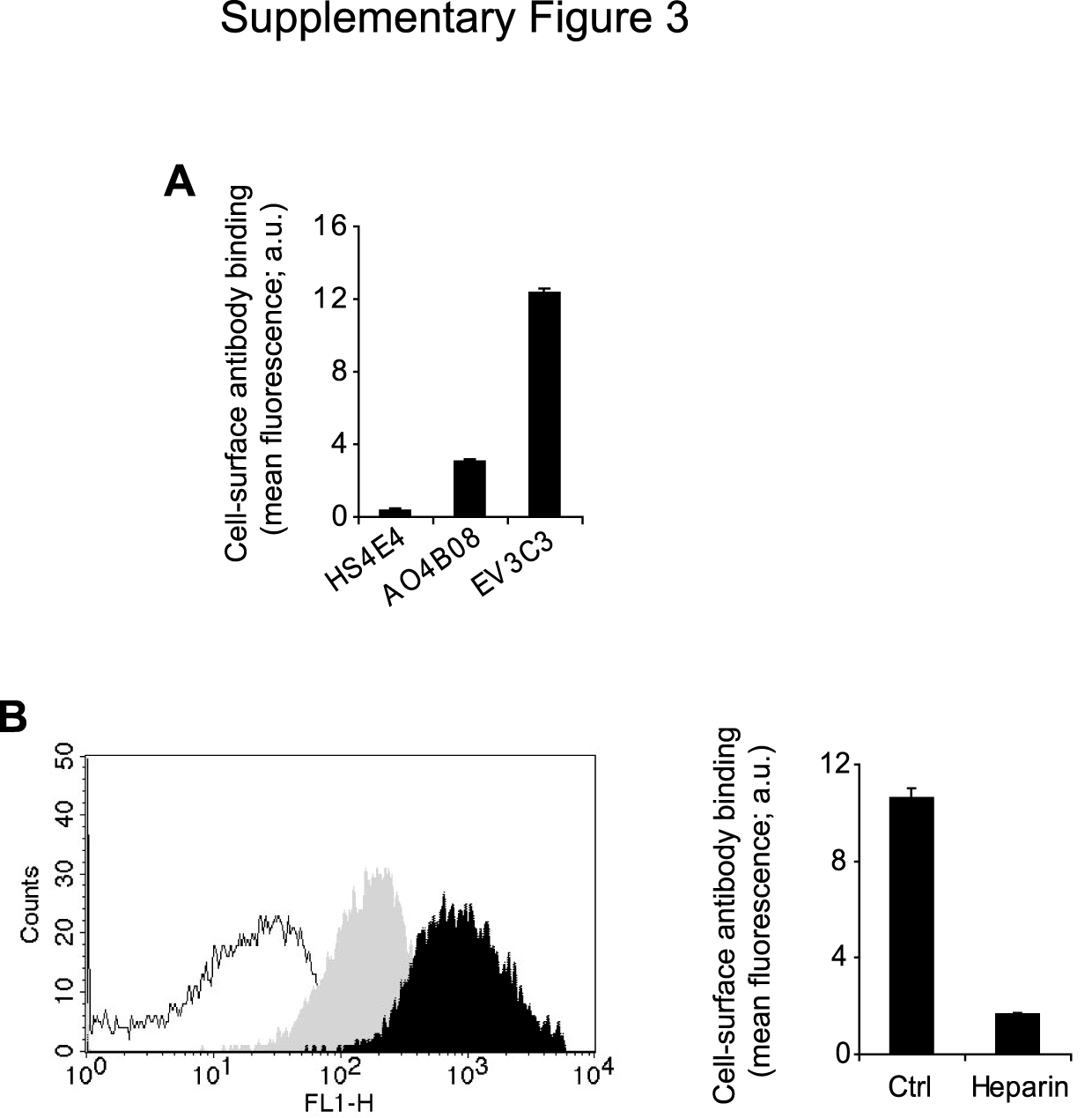

Supplement: Figure S3 — Differential binding of AO4B08, EV3C3, and HS4E4 αHS clones to ECs. A, HUVECs were detached by concentrated PBS (x2)/EDTA and surface stained at 4° with AO4B08, EV3C3, or HS4E4 αHS clones (10 µg/ml). αHS cell-surface binding was analysed by flow cytometry. B, HUVECs were surface stained at 4° with mouse anti-vsv antibody and Alexa Fluor 488-conjugated rabbit anti-mouse antibody (Ctrl, white area), or with vsv-tagged AO4B08, mouse anti-vsv antibody, and Alexa Fluor 488-conjugated rabbit anti-mouse antibody in the absence (black area) or in the presence of heparin (10 µg/ml; grey area). Cell surface binding was analysed using flow cytometry. Right panel: Data are presented as the average±S.D. (TIF) [file pone.0049092.s003.tif]

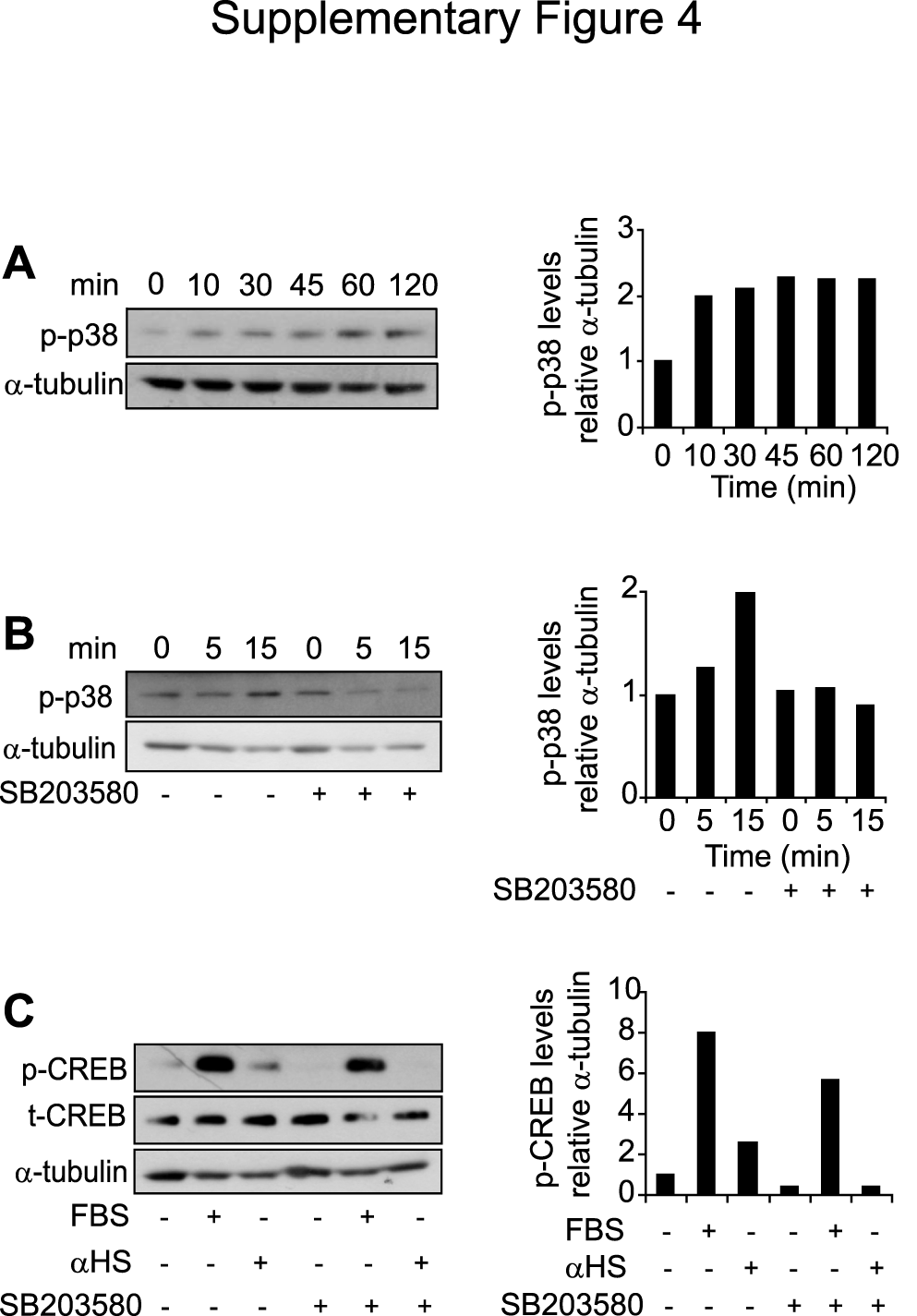

Supplement: Figure S4 — αHS induces P38 MAPK activation in U-87 MG cells and αHS-mediated signalling is blocked by P38 MAPK inhibition. A, U-87 MG glioblastoma cells were either untreated (Ctrl) or incubated with αHS (AO4B08; 1.25 µg/ml) for the indicated time periods followed by immunoblotting for phospho-p38 MAPK and α-tubulin. Left panel shows representative immunoblots of three separate experiments. Right panel shows quantification of relative phospho-p38 MAPK levels (p-p38/α-tubulin) from a representative experiment. B, Same experiment as in (A) was performed with HUVECs, either untreated (Ctrl) or treated with αHS (AO4B08; 1.25 µg/ml) for the indicated time periods with or without 30 min pre-treatment with p38 MAPK inhibitor SB203580 (2 µM) as indicated. Left panel shows representative immunoblots of three separate experiments. Right panel shows quantification of relative phospho-p38 MAPK levels (p-p38/α-tubulin) from a representative experiment. C, HUVECs were left untreated or pre-treated with p38 MAPK inhibitor SB203580 (2 µM) for 30 min, stimulated with FBS (10%) or αHS (AO4B08; 1.25 µg/ml), followed by immunoblotting for phospho-CREB, total CREB, and α-tubulin. Left panel shows representative immunoblots of three separate experiments. Right panel shows quantification of relative phospho-CREB levels (p-CREB/α-tubulin) from a representative experiment. (TIF) [file pone.0049092.s004.tif]
